# Treatment of carpal tunnel syndrome with wrist splinting: study protocol for a randomized placebo-controlled trial

**DOI:** 10.1186/s13063-019-3635-6

**Published:** 2019-08-27

**Authors:** Isam Atroshi, Kamelia Tadjerbashi, Steven J. McCabe, Jonas Ranstam

**Affiliations:** 1 Department of Clinical Sciences – Orthopedics, Lund University, SE-22100 Lund, Sweden; 2 Department of Orthopedics Hässleholm-Kristianstad, Hässleholm Hospital, SE-28125 Hässleholm, Sweden; 3 Department of Surgery, University of Toronto, Toronto Western Hospital, Toronto, ON M5T 2S8 Canada

**Keywords:** Carpal tunnel syndrome, CTS, Median nerve, Wrist, Non-surgical treatment, Splinting, Randomized trial, RCT

## Abstract

**Background:**

Carpal tunnel syndrome (CTS) is a common cause of pain, weakness, sensory loss, and activity limitations. Currently, the most common initial treatment is use of a rigid splint immobilizing the wrist, usually during night-time, for several weeks. Evidence regarding the efficacy and effect durability of wrist splinting is weak. The treatment is associated with costs and may cause discomfort and limit daily and work activities. No placebo-controlled trials have been performed.

**Methods:**

This is a randomized controlled trial designed to assess the efficacy of a rigid wrist splint compared with soft wrist bandage (placebo) in patients with primary idiopathic CTS. The trial will be conducted at an orthopedic department. Patients, 25 to 65 years old, who seek primary health-care with symptoms of CTS will be screened, and potentially eligible patients will be referred to the study center. Patients who fulfill the trial’s eligibility criteria will be invited to participate. A total of 112 patients who provide informed consent will be randomly assigned to treatment with either a rigid wrist splint or a soft bandage to be used initially for 6 weeks at night and, if possible, during the day. The splints and bandages will be fitted with a temperature-monitoring device to measure the total time during which they have actually been worn. The trial participants will complete a questionnaire that includes the 6-item CTS symptoms scale (CTS-6); the 11-item disabilities of the arm, shoulder, and hand (*Quick*DASH) scale; and the EuroQol 5-dimension (EQ-5D) health status and quality-of-life measure at baseline and at 6, 12, 24, and 52 weeks after treatment start. The participants will undergo physical examination and nerve conduction testing at baseline and at 52 weeks. The trial’s primary outcomes are the change in the CTS-6 score from baseline to 12 weeks and the rate of carpal tunnel release surgery at 52 weeks.

**Discussion:**

This is the first placebo-controlled randomized trial with electronic monitoring of actual splint use and will provide evidence regarding the efficacy of wrist splinting in patients with CTS.

**Trial registration:**

ISRCTN Registry, ISRCTN81836603. Registered on May 5, 2018.

**Electronic supplementary material:**

The online version of this article (10.1186/s13063-019-3635-6) contains supplementary material, which is available to authorized users.

## Background

Carpal tunnel syndrome (CTS) is a very common cause of hand pain, weakness, and loss of sensation leading to limitations in daily activities, work disability, and worsening quality of life [1]. The prevalences in the adult general population are about 5% among women and 2% among men [2]. The goal of treating CTS is to relieve symptoms and improve hand function. Currently, the most common non-surgical treatment across the world is splinting the wrist with a rigid splint, usually at night, sometimes combined with other treatments [3]. There is some evidence that wrist splinting may be effective in the short term [4–6], but the evidence is generally weak, the optimal duration of treatment is unclear, and the long-term efficacy has not been established [7, 8]. Treatment benefit of wrist splinting itself has often been small and of short duration, but in trials that compared splinting with surgery [5, 9], the benefit has often been augmented by large cross-over to surgery [10]. The rationale behind wrist splinting is that it prevents wrist flexion, known to increase pressure in the carpal tunnel [11]. However, the duration of splinting currently used in clinical practice (4 to 6 weeks) is not based on clear evidence [7]. The long-term effect of splinting the wrist for this short time on the pathophysiological factors involved in the causation of CTS may not be large. It is unclear why the benefit from wrist splinting in idiopathic CTS can persist after cessation of splinting.

In previous randomized studies, treatment of CTS with wrist splint has been compared with surgery [5] or other non-surgical treatments, such as steroid injection or exercises [7, 12]. Of the non-surgical treatments, only local steroid injection has strong evidence from placebo-controlled trials supporting short-term efficacy [12]. However, it is still an invasive procedure not routinely available in primary care and thus may require referral to specialists. The few randomized studies that evaluated wrist splinting in the treatment of patients with CTS were not placebo-controlled and therefore the reported improvement may have been related to non-specific effects or the natural course of the disease. Compliance in wearing the splint was often not evaluated or was assessed by asking patients to register data in a diary, a method with uncertain reliability. A previous study has shown that patients tend to overestimate their splint use [13].

Although splinting is a simple and safe treatment, it has some disadvantages. Patients may find that wearing a splint is uncomfortable and limits them in some work or daily activities or both. The costs of the splint and therapy visits may be high [14]. There is a need for a randomized placebo-controlled trial assessing the efficacy of wrist splinting in the treatment of patients with CTS.

### Trial objective

The objective of the trial is to evaluate the placebo-controlled treatment efficacy and effect durability of wrist splinting in patients with primary idiopathic CTS up to 12 months after treatment start. Our hypothesis is that, in patients with CTS, wearing a rigid wrist splint at night and, if possible, during the day for 6 weeks is more effective than wearing a soft wrist bandage in reducing symptoms and subsequent need for surgery.

## Methods

### Trial design and setting

The study is a prospective randomized parallel-group superiority clinical trial conducted at one university health-care orthopedic department (Department of Orthopedics, Hässleholm-Kristianstad-Ystad) in collaboration with several primary care centers in the region of Northeastern Skåne in southern Sweden (population of 300,000). The department is the only referral facility for patients with CTS in that region. The Standard Protocol Items: Recommendations for Interventional Trials (SPIRIT) checklist is provided as an Additional file 1.

### Inclusion criteria

- Primary, idiopathic CTS
- Age 25–65 years, either sex
- Symptoms of classic or probable CTS according to the criteria in the Katz hand diagram [15]
- Two surgeons (specialists in orthopedic or hand surgery) independently diagnose the patient’s CTS
- Symptom duration of at least 1 month

### Exclusion criteria

- CTS classified as severe (thenar muscle atrophy or 2-point discrimination exceeding 8 mm in at least one finger)
- Treatment of the study hand with a wrist splint in the past 12 months
- Previous steroid injection for CTS in the study hand
- Inflammatory joint disease
- Vibration-induced neuropathy
- Polyneuropathy
- Current pregnancy
- Trauma to the study hand in the past 12 months
- Previous CTS surgery in the study hand
- Inability to complete questionnaires because of language difficulties or cognitive disorder
- Severe medical illness
- Known abuse of drugs or alcohol or both

### Screening

Patients consulting a primary care physician or referred to occupational therapists at primary health-care centers for symptoms suggestive of CTS will be screened. Potentially eligible patients are referred to the orthopedic department and scheduled for assessment by two surgeons in the research team (a senior hand surgeon and an orthopedic specialist) within 1 to 2 weeks of referral. Both surgeons will be present when a full history is taken, but the following physical examination will be carried out by the orthopedic specialist only. Patients judged to fulfill the eligibility criteria are then given, by the orthopedic specialist, full verbal and written information about the aims and conduct of the trial as well as the potential advantages and disadvantages of participation. Patients who accept participation will provide written informed consent. Participants will undergo the baseline assessment immediately and nerve conduction testing as soon as possible but no later than 2 weeks after enrolment. Only one hand will be included in the trial (in bilateral symptoms, the hand with the worse score on the 6-item CTS symptoms scale (CTS-6) will be included). Each patient will be allowed to enter the trial only once.

### Randomization

**Fig. 1:**
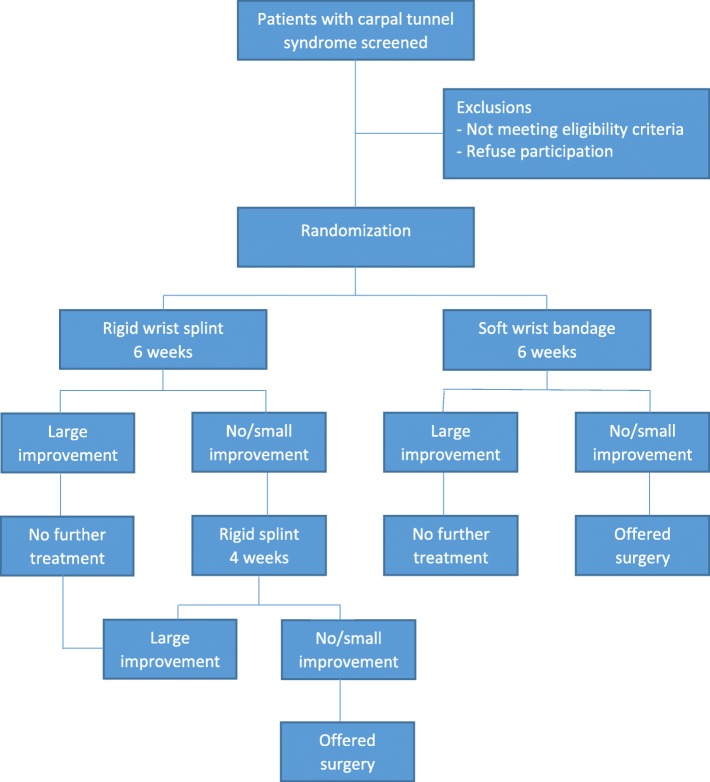
Patient flow through the trial

Patients will be randomly assigned in accordance with a computer-generated randomization list (ratio of 1:1) [16]. The randomization will be stratified in accordance with patient sex and carried out in random blocks of various sizes (4, 6, and 8). An administrative assistant, not involved in the trial, will prepare sequentially numbered sealed opaque envelopes containing the group allocation. After providing written informed consent and undergoing the baseline assessment by a study investigator (orthopedic surgeon), the enrolled patient will proceed to the hand therapist, who will open the envelope with the lowest number and provide the patient with either a wrist splint with a metal bar or a soft bandage in accordance with treatment allocation (Fig. 1).

### Interventions

#### Group A: Splint with metal bar

**Fig. 2:**
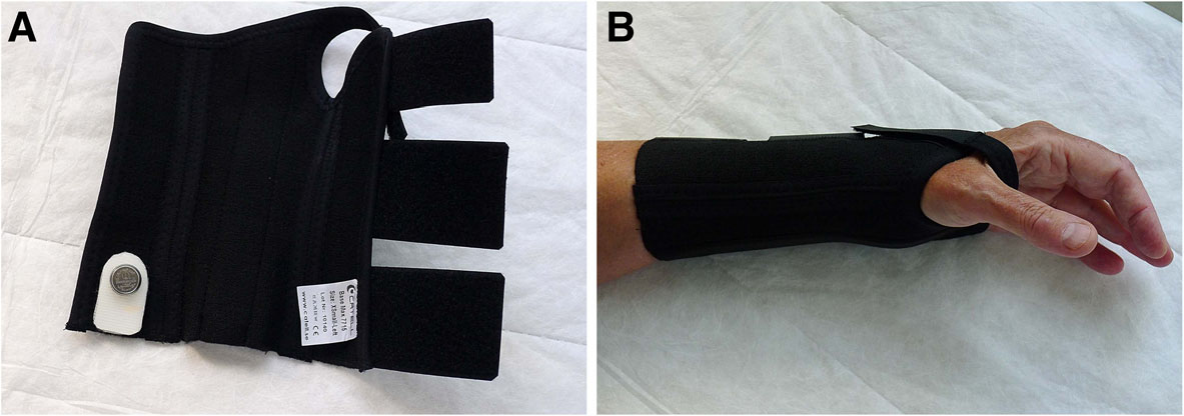
Conventional splint containing a metal bar and a temperature-monitoring device (**a**), holding the wrist in neutral position (**b**)

The patients will receive a standard splint (model Base, Catell AB, Hägersten, Sweden) with wrist in neutral position to be worn at night and, if possible, during the day (Fig. 2). No other instructions or treatments will be given. If after 6 weeks the patient reports large improvement, no further treatment will be given. If the patient reports small or no improvement, further treatment with the same splint will be given for 4 weeks. If the patient reports small or no improvement after 10-week splinting, the patient will be offered surgery. Patients who refuse further treatment with wrist splinting will be offered surgery. Surgery will not be performed before 12 weeks after treatment start.

If symptoms recur after improvement, the patients will be treated with 4 weeks of wrist splinting using the same type of splint. If after 4 weeks the patient reports small or no improvement, the patient will be offered surgery. Patients who refuse further treatment with wrist splinting will be offered surgery.

#### Group B: Soft bandage

**Fig. 3:**
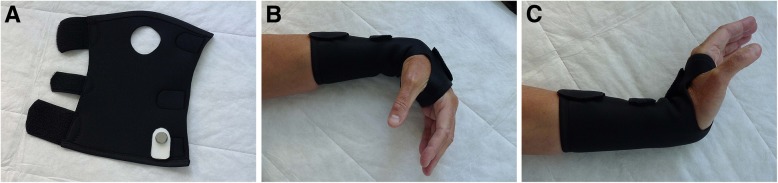
Soft bandage (**a**) allowing full wrist flexion (**b**) and extension (**c**)

The patients will receive a custom-made (neoprene) wrist bandage to be worn at night and, if possible, during the day (Fig. 3). No other instructions or treatments will be given. If after 6 weeks the patient reports large improvement, no further treatment will be given. If the patient reports small or no improvement, the patient will be offered surgery. Surgery will not be performed before 12 weeks after treatment start.

If symptoms recur after improvement, the patient will use the same type of bandage for 4 weeks. If after 4 weeks the patient reports small or no improvement, the patient will be offered surgery. Patients who refuse further treatment with the wrist bandage will be offered surgery.

#### Discontinuing/modifying allocated interventions

The trial interventions (wrist splint or soft bandage) are not expected to cause harms, but it is possible that a participant will experience discomfort using a splint or bandage. Participants will be asked to continue their allocated intervention if possible. The participants are informed that, in case of worsening of symptoms during intervention, they contact the trial therapist, who will discuss the case with the investigators. If the worsening is not experienced by the participant as severe, the participant will be asked to continue the allocated intervention in accordance with the protocol. If symptoms are severe or the participant declines to continue the allocated treatment, surgery will be offered. The participant will be asked to continue the allocated treatment until surgery.

#### Concomitant care

No other treatments will be prescribed during the trial interventions. The information provided to participants will not specify any prohibitions. Participants will be able to take non-prescription analgesics. Cross-over between the trial interventions is not allowed.

### Follow-up procedures

**Fig. 4:**
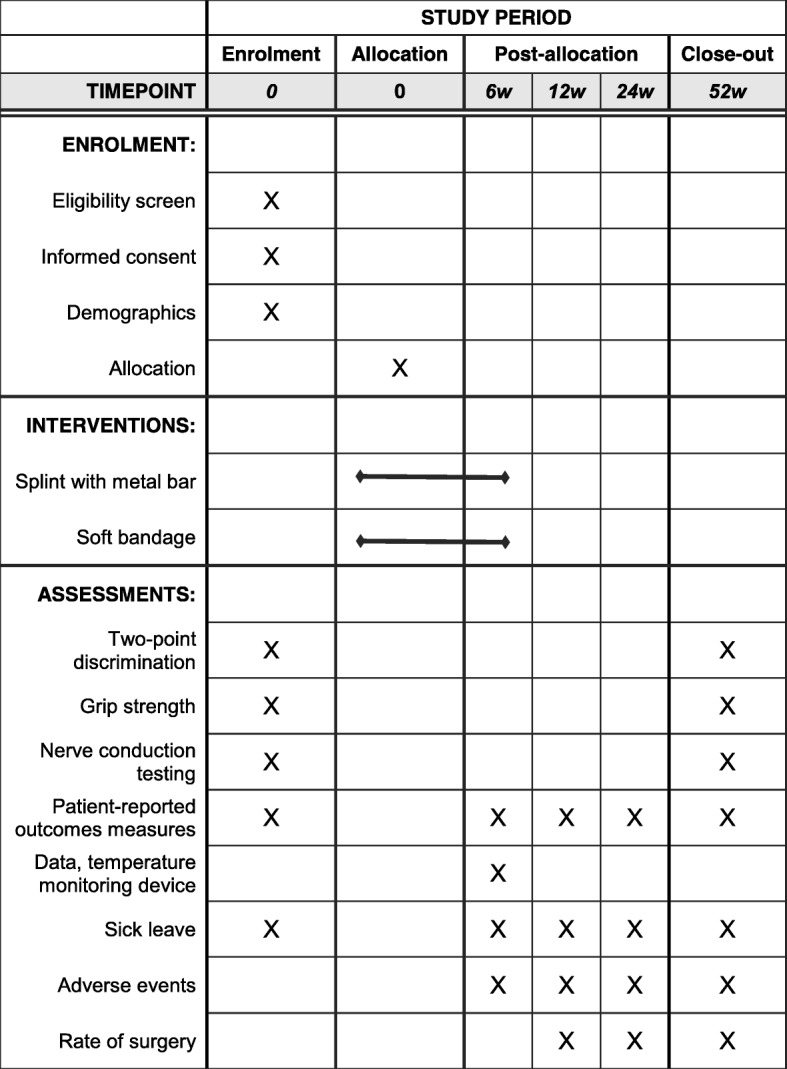
The schedule of enrolment, interventions, and assessments

Patients will complete a questionnaire consisting of disease-specific and generic patient-reported outcome measures at baseline and at 6, 12, 24, and 52 weeks after treatment start and will attend physical examination and nerve conduction testing at 52 weeks (Fig. 4). The trial coordinator (experienced research nurse) will monitor completeness of questionnaires and use telephone contact when necessary. Participants who choose to undergo surgery will be asked to complete the questionnaire shortly before surgery if the date of surgery precedes or is more than 2 weeks after the scheduled follow-up dates. Participants will be informed, in writing and during examination/follow-up, about the importance of completing the intervention and the follow-up procedures. Patients who choose to discontinue intervention are asked to, if possible, respond to the outcome questionnaires at the intervals defined in the protocol and attend the 1-year follow-up.

#### Outcome measures

The CTS-6 is a validated questionnaire inquiring about severity, frequency, and duration of symptoms—including nocturnal and daytime pain and numbness or tingling—experienced by the patient in the past two weeks [17]. Each item has five possible response options, which range from 1 (no symptom) to 5 (most severe symptom). The symptom score is the mean of all answered items; higher scores (1 to 5) indicate worse symptoms. The *Quick*DASH is a short form of the disabilities of the arm, shoulder, and hand (DASH) questionnaire, a widely used outcome measure for upper extremity disorders, including CTS [18]. The *Quick*DASH consists of 11 items concerning difficulties in performing activities with five response options (from no difficulty to unable to perform an activity). Higher score (0 to 100) indicates worse activity limitations. The EuroQol 5-dimensions (EQ-5D) is a widely used measure of general health and quality of life; the EQ-5D-5 L version will be used [19]. The palmar pain scale is a two-item scale measuring pain in the palm and related activity limitations; higher score (0 to 100) indicates more pain and activity limitations [20]. Treatment satisfaction will be measured with a visual analog scale asking the patients to rate (on a scale of 0 to 100) their satisfaction with their current hand status with regard to symptoms and ability to use it in daily activities (higher score indicates higher satisfaction).

#### Physical examination

The physical examinations will be performed by an orthopedic specialist and will include measurement of 2-point discrimination (performed on the radial and ulnar aspects of each digit) and measuring grip strength with the Jamar dynamometer and pinch strength with the pinch gauge (three trials for each hand). The examiner will be blinded to group allocation, and at the 12-month follow-up, the palm will be covered to conceal possible surgical scars.

#### Nerve conduction tests

Median nerve conduction testing of the study hand will be carried out by a trained research nurse and interpreted by a neurophysiologist, both blinded to the clinical status. The measurements include median nerve distal motor latency and wrist-digit distal sensory latency in the index finger (median nerve), ring finger (median and ulnar nerves), and small finger (ulnar nerve). The results are classified as normal or as mild, moderate, or severe median neuropathy (Table 1) in accordance with standard neurophysiological criteria [21, 22].

**Table 1:** Classification of nerve conduction testing results

Grade	Motor latency^a^	Sensory latency^b^	
		Peak latency difference (index-small)^c^	Inter-peak latency (ring)
Normal	Normal	<0.6	Single peak
Mild	Normal	≥0.6	≥0.6
Moderate	Abnormal	>1.2	>1.0
Severe	Abnormal	Absent response	Absent response

All values are in milliseconds. A result is classified as abnormal if one or more criteria are present

^a^Distal motor latency values according to age-based reference limits. (For example, values of more than 4.2 ms for age of 30 years and of more than 4.3 ms for age of 50 years are considered abnormal.)

^b^Median-ulnar sensory latency difference

^c^Index finger peak latency values above 3.8 ms (women) and 4.0 ms (men) are considered abnormal

#### Adherence

In written information before randomization and during all contacts with the research team, the trial participants will be informed about the importance of adhering to the allocated intervention.

#### Measurement of actual splint and bandage use

Both the rigid splint and the soft bandage will be fitted with a temperature-monitoring device that registers temperature variations according to whether the splint or bandage is in contact with the skin (Fig. 2). The Thermochron^®^ iButton^®^ device (Maxim Integrated, San Jose, CA, USA) is a small disk recording the temperature at predefined intervals and stores the results in a protected memory section. This type of temperature sensor is commonly used to monitor the cold chain of the food industry and also for pharmaceutical and medical products; a similar device has been previously used in clinical research [23, 24]. The device records temperature ranging from −40 °C to 85 °C with measurement accuracy of 1 °C. In this study, the recording intervals will be set at 40 min. After 6 weeks of splint use, the disc will be removed and the measurements will be uploaded to a computer. At the conclusion of the trial, the duration and pattern of wearing the splint will be computed and analyzed.

#### Surgery

The decision to choose to have surgery will be made by the patient, on the basis of the experienced severity of current symptoms and activity limitations, in consultation with an orthopedic surgeon not involved in the trial and blinded to the patient’s group allocation. Surgery will be performed by specialists in orthopedics or hand surgery not involved in the trial, in accordance with usual practice at the department.

### Assessments of efficacy

#### Primary endpoints (in rank order)

1. Change in the 6-item CTS symptoms score from baseline to 12 weeks
2. Rate of surgery at 52 weeks

#### Secondary endpoints

1. Change in the 6-item CTS symptoms score from baseline to 6 weeks and 52 weeks
2. Change in *Quick*DASH score from baseline to 12 weeks and 52 weeks
3. Change in patient satisfaction score at 12 weeks and 52 weeks
4. Change in EQ-5D index from baseline to 12 weeks and 52 weeks
5. Cost-effectiveness at 52 weeks
6. Palmar pain score at 52 weeks
7. Time to surgery within 52 weeks
8. Duration of sick leave during 52 weeks
9. Change in grip strength from baseline to 52 weeks
10. Adverse events at 52 weeks

### Assessment of safety

During the conduct of the trial, the investigator will report all adverse events. All adverse events will be followed up until resolved or as clinically required. Adverse events may be reported spontaneously by the patient or elicited through open questioning during and at the end of the trial. Participants will be informed to contact the trial therapists or coordinator whenever they wish to discuss or report any events during the intervention. In addition, they will be able to report adverse events at the 6-week follow-up and any subsequent follow-up. All reported or observed adverse events, including type, intensity, and duration, will be recorded in a standard protocol. Any serious adverse events will be promptly reported to the steering and data monitoring committees and the trial sponsor.

### Assessment of costs

The costs of the rigid splint and soft bandage and the visits to therapist will be calculated. Cost for sick leave will be estimated by multiplying the number of days by the estimated mean income for the profession of that patient. In case of unemployment, a fixed rate will be used and for patients on maternity leave, cost will be calculated as for the working patients.

### Staff information and documentation

All personnel involved with the trial will be informed orally and in writing about the trial procedures. Case report forms (CRFs) will be provided for recording of all data. Accuracy of the data on each CRF will be attested by the examiner’s signature. If any assessments are omitted, the reasons will be noted on the CRFs. The patients’ responses to questionnaires will be checked for completeness by the examiner or trial coordinator. All data will be stored in the department’s databases accessed only by the trial researchers.

### Monitoring and data management

The trial coordinator will keep all records related to randomly assigned participants at the research unit. The trial will be monitored by an independent three-member data monitoring committee (a senior orthopedic surgeon with experience in clinical research and two trained research nurses). A committee member will regularly (on a monthly basis) examine the records to ensure that the conduct of the trial and data collection is in accordance with the trial protocol. The steering committee will comprise the two on-site investigators, trial coordinator, and members of the data monitoring committee. The data management team will include the trial’s principal investigator (IA), a co-investigator (KT), and a statistician (JR).

### Withdrawals

Patients will be able to withdraw from the trial at any time without need to give reasons. Patients who do not wish to attend physical examination will be asked to complete the questionnaire.

### Sample size

In a previous study, patients with idiopathic CTS improved on average by 1.6 points in the CTS-6 score (range of 1 to 5) at 12 weeks after surgery [20]. No previous data regarding change in CTS-6 score after use of wrist splinting or soft bandage are available. A score improvement of 0.7 corresponds to improvement by one severity level in four of the six items (i.e., from severe to moderate, moderate to mild, or mild to no symptoms, in more than half of the items). If the soft bandage is assumed to have no effect, it would correspond to “no treatment”. A previous study found that the mean change in CTS-6 score in a group of patients who completed the CTS-6 on two occasions with 1–3 weeks’ interval *without treatment* was 0.03 (95% confidence interval (CI) of −0.07 to 0.12) [17]. With 90% power, 5% significance level, two-sided tests, and mean changes (baseline to 12 weeks) in the CTS-6 score of 0.7 in the splint group (standard deviation of 0.9) and 0.1 in the soft-bandage group, a sample of 48 patients per group will be needed. To account for possible dropouts, we plan to recruit 112 patients.

### Statistical analysis

For continuous endpoints (CTS-6, *Quick*DASH, patients satisfaction, palmar pain, EQ-5D index, grip strength, time to surgery, and sick leave duration), mean values and standard deviations will be calculated. For categorical variables (rate of surgery and adverse events), proportions will be calculated. Statistical tests will be performed in accordance with the intention-to-treat principle. An exploratory as-treated analysis will also be performed.

Both hypothesis-generating and confirmatory testing will be performed, the latter for the primary endpoints. Multiplicity issues will be addressed in compliance with the European Medicines Agency’s Guideline on multiplicity issues in clinical trials. More specifically, the primary endpoints are ranked according to clinical relevance, and confirmatory claims will not be based on an endpoint with a rank lower than the variable whose null hypothesis was the first that could not be rejected. The subgroup analyses will be carried out in rank order.

#### Primary analyses

The change in CTS-6 score from baseline to 12 weeks (primary outcome) will be compared in the two groups by using mixed model analysis of repeated measures and adjusting for the baseline score. The rate of surgery at 52 weeks (co-primary outcome) will be compared by using Cox regression analysis with fixed follow-up time and the Huber–White estimator [25, 26] and adjusting for age, dominance of the study hand, and baseline CTS-6 score; relative risks with 95% CIs will be calculated. As supportive analysis the chi-squared test will also be performed.

#### Secondary analyses

Mean changes in *Quick*DASH score, EQ-5D index, and grip strength over time (from baseline to 52 weeks) will be compared by using mixed model analysis of repeated measures and adjusting for respective baseline values. Mean treatment satisfaction and palmar pain scores at 12 and 52 weeks will be compared between the groups by using the *t* test. Mean total duration of sick leave from treatment start to 52 weeks will be calculated and compared by using Satterthwaite’s *t* test. Time to surgery (in days) will be analyzed by constructing Kaplan–Meier curves and comparing the groups with the log-rank test. Cost-effectiveness will be analyzed by using the incremental cost-effectiveness ratio. Three subgroup analyses will be carried out (in rank order): baseline CTS-6 score (≥3.0 versus <3.0), baseline nerve conduction results (severe/moderate versus mild/normal), and symptom duration (≥6 versus <6 months). Adverse events will be presented in tables. A *P* value of 0.05 will indicate statistical significance.

### Missing values

For the patient-reported measures, missing item responses will be managed in accordance with the instructions specific to each scale. If the number of missing items precludes calculating a score, the missing score will not be replaced. Missing values for other variables will not be replaced.

### Blinding

Blinding of patients to type of treatment is not possible. The primary outcome is a patient-reported outcome measure. Baseline and follow-up examinations, including nerve conduction tests, will be carried out by blinded assessors. Analysis of splint/bandage use data will be carried out by a blinded analyst. Discussions with patients about possible surgery and all possible surgical procedures will be performed by blinded surgeons. Interpretation of nerve conduction tests will be carried out by a blinded neurophysiologist. All statistical analyses will be carried out by a blinded statistician.

### Ethics

The trial has been approved by the regional ethical review board (reference number: 2018/16; date: January 30, 2018). The trial will be conducted in accordance with the Declaration of Helsinki.

### Recruitment strategy and timeline

Participants are recruited through referrals from primary care physicians and occupational therapists. Written information about the trial has been given (via e-mail) to all primary care units in the study region. To enhance recruitment, meetings with primary care therapists during which further information was given were held. Recruitment is expected to be completed in 2 to 3 years. If, during the trial, recruitment strengthening is deemed necessary, other strategies will be considered and discussed with the steering committee and approval from the ethical review board will be sought. No financial or non-financial incentives are provided to the trial participants (except for their contribution to the research’s potential future benefit to patients with this condition).

### Protocol modifications

Any important protocol modifications will first be presented to the ethical review board for approval and then communicated to relevant parties, including trial investigators, primary care physicians/occupational therapists, and involved participants.

## Discussion

CTS is a very common condition affecting millions of people around the world [27]. Despite weak evidence, wrist splinting, alone or in combination with a variety of treatments, is currently the most common non-surgical treatment around the world.

A few previous studies have evaluated wrist splinting in the treatment of CTS by using the 11-item symptom severity scale (Boston CTS questionnaire), a scale that corresponds to the CTS-6, to measure symptoms [17]. A previous study that came closest to a placebo-controlled design compared two types of splints: a conventional rigid wrist splint (*n* = 46) and a soft splint (*n* = 45) that limits motion of the metacarpophalangeal joints but does not immobilize the wrist (although it is unclear whether full flexion was possible) [28]. The mean symptom severity score at baseline was 2.9 in both groups, and despite a small to moderate improvement at 3 months, the results at 9 months showed only a small mean score change (0.4 and 0.3, respectively), a difference of uncertain clinical importance.

In a study comparing steroid injection with 1-month night-time wrist splinting [29], the baseline mean symptom severity score in the splint group (*n* = 25) improved by 0.38 (standard deviation of 0.5) at 8 months (from a relatively low mean baseline score of 2.0). In another study comparing platelet-rich plasma injection with 6-month night-time wrist splinting, the mean symptom severity scores in the splint group (*n* = 60) were 1.7 at baseline and 1.5 at 6 months [30]. In a randomized study that compared electroacupuncture with 4-month night-time wrist splinting [31], the mean symptom severity score in the splint group (*n* = 91) was 2.4 at baseline and had changed by only 0.09 at 4 months.

In a study comparing ultrasound-guided pulsed radiofrequency with 12-week night-time wrist splinting, the mean symptom severity score in the splint group (*n* = 18) improved from 3.0 at baseline to 2.0 at 12 weeks [32]. Thus, although the studies assessing wrist splinting alone have reported conflicting results, the majority have shown small changes in the symptom severity score even with splinting longer than the time used in clinical practice.

A previous study from the Netherlands suggested that surgery was more cost-effective than wrist splinting in the treatment of CTS [14]. Although it is well established that carpal tunnel release is effective in treating CTS with good long-term results [33], it has several disadvantages, including surgery-related pain and hand weakness that may last several months after surgery [34]. In addition, surgery is associated with direct costs as well as indirect costs related to work absence after surgery [35].

In the diagnosis of CTS, the history (including type and characteristics of the symptoms, their distribution in the hand, and presence or absence of other concurrent arm symptoms) is of the utmost importance. Clinical examination might be helpful but usually does not compensate when the history is not strongly indicative of CTS. Physical examination is important in establishing the presence of any exclusion criteria, such as thenar muscle atrophy and abnormal 2-point discrimination. The trial will not demand positive provocative tests (Tinel sign and Phalen test) for the diagnosis, although these will be part of the clinical examination. Two surgeons will be involved in the screening; if either judges that the history does not clearly suggest a CTS diagnosis, the patient will not be included. If the subsequent physical examination, performed by one of the surgeons, reveals the presence of any exclusion criteria, the patient will not be included. Besides, median nerve conduction tests will be performed at baseline, although inclusion will not require abnormal test results. However, baseline nerve conduction test results are important when the results of the trial are reported because they describe the characteristics of the trial participants in terms of disease severity. This trial has more stringent eligibility criteria than many previous clinical trials; this is important because an incorrect diagnosis compromises the assessment of the intervention’s efficacy.

In a recent pragmatic primary care multicenter trial that compared wrist splinting with local steroid injection in CTS, the diagnosis was made by one of many different clinicians (doctors or therapists), and no nerve conduction tests were performed [36]. In the wrist-splinting group, the mean symptom severity score improved (from baseline) by 0.48 at 6 weeks and 0.73 at 6 months. The latter value is similar to the value considered a clinically important CTS-6 score change in the sample size calculation in our trial.

To our knowledge, this will be the first randomized placebo-controlled trial that evaluates the efficacy of wrist splinting in patients with CTS and the first to use an electronic monitoring device to measure time of active splint use. The evidence generated from this randomized trial can be expected to have large significance for patients and society.

## Trial status

Recruitment started June 4, 2018, and is expected to conclude by the end of 2020.

## Protocol version and date

This protocol is version 2.1 (dated July 31, 2019). No amendments have been made after the first patient was enrolled. Any subsequent amendment will be reported to the registry.

### Dissemination

The trial results will be communicated to the participants and published in a scientific journal.

## Additional file

Additional file 1: SPIRIT (Standard Protocol Items: Recommendations for Interventional Trials) Checklist*. (DOC 122 kb)

## Data Availability

The data will be available upon reasonable request.

## Abbreviations

CI: Confidence interval
CRF: Case report form
CTS: Carpal tunnel syndrome
CTS-6: 6-item carpal tunnel syndrome symptom score
EQ-5D: EuroQol 5-dimensions
*Quick*DASH: 11-item disabilities of the arm, shoulder, and hand
